# Opioid Detection Using Smartphone-Based Eye-Scanning

**DOI:** 10.3390/s25175467

**Published:** 2025-09-03

**Authors:** Kiki W. K. Kuijpers, Karl Andersson, Albert Dahan, Markku D. Hämäläinen, Monique van Velzen

**Affiliations:** 1 Department of Anesthesiology, Leiden University Medical Center, 2333 ZA Leiden, The Netherlands; 2 Skillsta Teknik Design och Kvalitet AB, Vänge, SE75578 Uppsala, Sweden; karl@skillsta.com; 3 Digital Health Research, Skövde University, SE54128 Skövde, Sweden; 4 Centre for Human Drug Research, 2333 CL Leiden, The Netherlands; 5 Outcomes Research Consortium, Houston, TX 77030, USA; 6 MediD Consultancy Group, 1077 XD Amsterdam, The Netherlands; 7 Kontigo Care AB, SE75320 Uppsala, Sweden

**Keywords:** oxycodone, opioid, detection, eye-scanning, pupil-size, miosis

## Abstract

**Highlights:**

**What are the main findings?**

- Smartphone-based eye scanning can detect opioid use without individualization up to 5 h after intake.
- For smartphone-based eye scanning, there is no need for special lighting equipment.

**What is the implication of the main finding?**

- Consumer electronics is sufficiently good for high-quality pupillometry.
- Smartphone-based eye scanning can be the future for detecting opioid consumption.

**Abstract:**

Opioids are known to constrict pupils, and mobile phone-based self-administered eye scanning (MPSES) offers a potential method for monitoring opioid use in real-world settings. A clinical trial with 12 volunteers measured pupil size using MPSES under different light conditions (approx. 50 or approx. 500 lux) in the lab and over a week at home. Each participant made approximately 21 home tests, 12 in the lab without oxycodone and 16 in the lab after oxycodone intake. At the second visit the participants received a single dose of 20 mg oxycodone, and their pupil size was monitored hourly for 5 h. The pupil size after oxycodone intake was compared to drug-naïve tests performed at the lab and at home. Logistic regression models were built using measured pupil size and light conditions measured by the phone during each test, and a dichotomous variable indicating tests before or after oxycodone dosing as the outcome. The model demonstrated high classification accuracy (AUC = 0.94), with 82% true positives, 9% false positives, 91% true negatives and 18% false negatives. Misclassifications were largely due to difficulties measuring pupil size in individuals with corneal arcus, causing most of the false positive findings, and other interindividual differences. This shows that MPSES, including monitoring of ambient light conditions, can effectively detect opioid use within the 50–500 lux range. Our study paves the way for using MPSES to detect opioid use.

## 1. Introduction

Opioids, a class of drugs used primarily for pain relief, have a well-documented effect on pupil size [1]. When opioids bind to opioid receptors in the brain, they inhibit the release of neurotransmitters that would normally signal the pupils to dilate in response to low-light conditions [2]. This results in miosis, or pinpoint pupils, which is one of the hallmark signs of opioid use [2,3].

The pupil is an opening in the iris that controls the amount of light reaching the retina, hereby optimizing the quality of visual information [4]. In adults, pupil size varies considerably, ranging from 2–4 mm in bright light to 4–8 mm in the dark [5]. The pupil size is controlled by muscles in the iris. The sphincter pupillae causes miosis, for example, by the activation of the parasympathetic nervous system, and dilator pupillae enlarges the pupil causing mydriasis by the activation of the sympathetic nervous system [4].

Measuring pupil size can be a useful non-invasive method for assessing opioid consumption. Classically, penlight or ruler examination was performed to assess pupil size and light response [6]. Nowadays, manual or digital pupillometers are most used as handheld medical devices measuring the diameter of the pupil [7]. Most frequently, pupillary light reflex (PLR) measurements are performed, where the speed and extent of the pupil’s reaction to changes in light intensity is quantified. Under the influence of opioids, pupils typically constrict rapidly and remain small, even when the surrounding light decreases [8]. In a clinical or rehabilitative setting, regular monitoring of pupil size may help in assessing sobriety and compliance with opioid tapering protocols. The major limitation of penlight examinations is that they rely on visual assessment which can introduce variability and subjectivity, particularly if used by less experienced health care professionals [9].

One potential way to assess opioid consumption that does not rely on visual assessment by humans and not only measures pupil size but also detects opioid usage is a mobile phone-based eye-scanning technique (MPSES). Technological advancements have led to the development of MPSES which offers a convenient and efficient way to measure pupil size and detect opioid use. Mobile applications, apps, use the phone’s camera and built-in sensors to measure pupil size and the amount of light in the room [10]. During the development of these apps, measurements are mostly conducted in a consistent and controlled lighting environment, ensuring proper lighting conditions [10]. While these apps offer accessible, non-invasive, continuous monitoring options, the handling of variable lighting conditions may be of concern. The external light may lead to misinterpretation of pupil size and the pupil’s response to light, leading to false positives or false negative results when detecting opioid use [11]. Such apps may therefore be less suitable to detect opioid use ‘in the wild’. MPSES can be used in addiction care where it allows for patients to be monitored more regularly in an outpatient setting. This gives the patient more freedom while being more closely monitored, since there is no need for supervised urine tests.

Previous analyses of our study data, based on the statistical evaluation plan of the clinical study, used individualized data from one visit [10]. With individualization, a baseline measurement is made in a verified sober state. This baseline measurement is used for signal individualization which helps to detect drug use in a later test by accounting for the natural variation between individuals, such as a large pupil size.

In this reanalysis, we focused on detecting oxycodone without individualization with the goal of using mobile phones for general drug sobriety control. We utilized all drug-naïve baseline eye tests from both lab visits and all home tests from all 12 participants, comparing them to replicated tests taken up to 5 h after oxycodone administration. This approach increased the complexity by incorporating variations in ambient light, as tests were conducted in real-life conditions at home and at two controlled light settings in the lab. When scanning the eyes, the mobile phone first estimates the ambient light, after which its camera measures the pupil size before and after illumination. The combined data on pupil size and light conditions was used to develop a drug detection classifier, where the model distinguishes sober individuals from those under the influence of an opioid.

## 2. Methods

### 2.1. Study Design and Ethics

This study had a randomized, parallel, open-label feasibility design and took place in a single center in the Netherlands [10]. The main objectives of the study were to collect data with consumer-grade electronics (smartphones) and compare the findings with claims in the literature made using precision equipment. This study was conducted at the Anesthesia & Pain Research Unit of the department of Anesthesiology, Leiden University Medical Center, between February and July 2023, after receiving approval from the Ethics Review Board METC-LDD (Leiden, The Netherlands). All study procedures were performed according to good clinical practice guidelines and adhered to the tenets of the Declaration of Helsinki. This study was registered in the public trial register clinicaltrials.gov with identifier NCT05731999. Prior to study enrollment, all participants provided written informed consent.

### 2.2. Participants

Healthy male and female volunteers aged 18–70 years with body mass index 18.5–30 kg/m^2^ and weight 50–100 kg were eligible. People able to become pregnant had to provide a negative urine pregnancy test at enrollment and prior to the second study visit. Additional inclusion criteria were as follows: healthy based on medical history and physical examination at screening, no current drug use as defined by negative urine drug tests and able to use the MPSES app after initial training. Exclusion criteria were as follows: blind, deaf, pregnant or lactating, clinically relevant abnormal electrocardiogram at enrollment, current or previous alcohol abuse (AUDIT questionnaire), current or history of having a psychiatric disorder (MINI questionnaire), current treatment with medications that may affect eye measurements, or a known allergy for the drugs used in the study.

Participants were randomized based on self-reported eye color (light: blue/green/gray or dark: brown/black) and age category (18–20 years, 50–70 years, or 18–70 years) into one of four treatment groups (12 participants in each group). Each group contained at least 3 subjects with a light eye color and at least 3 subjects with a dark eye color, 2 subjects aged 18–25 years and 2 subjects aged 50–70 years, in a weighted fashion.

### 2.3. Data Collection

Eye characteristic data was collected with the Previct Drugs app embedded in the Previct platform (version 2.18, Previct Drugs; Kontigo Care AB, Uppsala, Sweden), which we denote as the MPSES app. This app is commercially available.

To capture the videos of both eyes, the participants were provided with either an iPhone 13 mini (Apple Inc., Cupertino, CA, USA) or Samsung S22 (Samsung Electronics Co., Yeongtong-gu, Suwon, Republic of Korea) smartphone. After training on how to use the MPSES app during the initial visit, video recordings of eye characteristics were made by the participants themselves at home (7 days ± 2) and during both visits to the research unit.

The MPSES app collects data using the default camera settings in the smartphone (30 frames per second, auto exposure, no calibrations required) from three different eye-scanning procedures: non-convergence, horizontal nystagmus and pupillary light reflex (Appendix A, Table A1). Pupillary light reflex data was collected using the back camera to allow the use of the flashlight of the phone, meaning that the subject had to rotate the phone and position it centered at a ~30 cm distance from the face through digitized voice guidance [12]. Voice guidance was in Dutch, a language in which all participants were proficient. Non-convergence and horizontal nystagmus were collected using the front camera at a ~25 cm distance, also performed with the help of digitized voice guidance. Ambient light conditions were estimated by the MPSES app prior to each eye-scanning procedure. A “test” refers to completion of all three of these procedures. A test took approximately 5–10 min.

**Figure A1:**
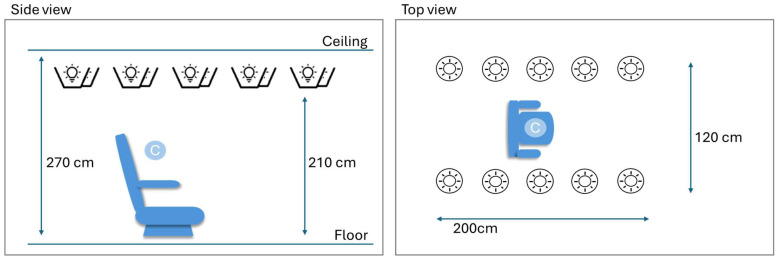
Description of the measurement area. Ten light sources were mounted facing upwards near the ceiling. Light sources could be controlled in terms of intensity. Two fixed intensity levels were created, producing either 50 lux or 500 lux at calibration position C.

The measurements made in the research unit were collected at two controlled light conditions, ~50 and ~500 lux. This corresponds to dim indoor lights and bright indoor lights, respectively. These conditions were created in a dedicated no-daylight room using smart controllable lighting equipment (IKEA) with 10 lamps (each 1055 lumen) mounted near the ceiling. Lamps were mounted in two rows forming a rectangle (see Appendix A, Figure A1) and illuminated the ceiling to produce indirect light. Light conditions were validated using a luminometer (Sekonic Flashmate L-308, Sekonic Inc., North White Plains, NC, USA) in the center position, where a participant would sit on a medical recliner chair (see Appendix A, Figure A1).

**Figure A2:**
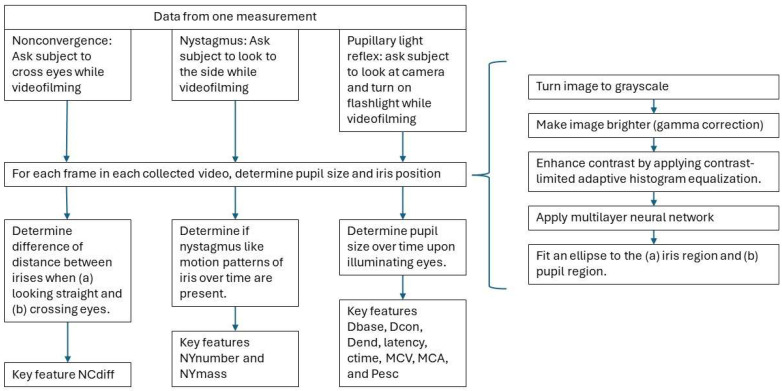
Processing of eye measurements. For each measurement (non-convergence, nystagmus and pupillary light reflex), the pupil size and iris position are determined. These are determined by turning the image to grayscale, brightening the image (gamma correction), enhancing contrast by applying contrast-limited adaptive histogram equalization and applying a multilayer neural network after which an ellipse is fitted to the iris region and to the pupil region. Based on the determined position, the key features are calculated.

The position of the iris and the size of the pupil were evaluated for each frame in the collected video recordings. This was performed by turning the image to grayscale, brightening the image (using gamma correction) and enhancing the contrast by applying contrast-limited adaptive histogram equalization (see Appendix A, Figure A2). After this a multilayer neural network (AI-model) was applied and an ellipse was fitted to the iris and pupil region. Using mathematical algorithms, the pupil size and eye movements (non-convergence/nystagmus (NC/NY)) were determined, which were used to calculate the key features. These key features were transformed into z-scores (Z-pupil size) by mean-centering and dividing by the standard deviation, based on sober data from all 12 participants who performed measurements before drug intake. Ambient light levels, measured using the mobile phone camera sensor, were expressed as the base-10 logarithm (Log10) of the light intensity measured by the phone.

During the first visit, participants performed 3 tests in each light condition. Following this visit the participants used the app daily at home for a week. Next, a full day session was conducted. For this visit, participants arrived at the research unit having fasted for at least 6 h. Upon arrival a urine drug and pregnancy test was performed, an intravenous access line was placed and three baseline measurements were made in each light condition. Thereafter, participants were administered the medicinal product: oxycodone (20 mg oxycodone HCl Teva, oral immediate release), or one of the three other drugs not discussed in this publication. Oxycodone was orally ingested with 100 mL non-carbonated water. Following drug administration, ocular characteristic data was collected from subjects at hourly intervals for a duration of 5 h post administration. After conducting eye scanning, a rapid quality control of the measurement was conducted via the app on the phone. If the test was invalid (due to excessive blinking or an inability to identify the iris), the test was repeated if time permitted.

Venous blood samples were drawn from the intravenous access line at the same time points as the eye measurements to allow for pharmacokinetic analyses (Ardena Bioanalysis, Assen, The Netherlands).

### 2.4. Data Analysis

The MPSES app converted collected data into key features [10]. For pupillary light reflex (PLR), key features essentially as illustrated by Hall and Chillcott were extracted [13].

A classifier for determining if an opioid had been ingested was made using logistic regression with the x-variables z-pupil size (z-score of the pupil size before ignition of the smartphone’s torch, Dbase) and log10 light measured and the y-variable OpioidIsOn (=1 after opioid ingestion, 0 otherwise) using JMP-Pro 16.2.0-statistical software (SAS Institute Inc, Cary, NC, USA).

## 3. Results

### 3.1. Participants and Recruitment

In total, 57 participants were screened. Six participants were excluded for not meeting eligibility criteria: two participants were excluded based on a positive drug test, one due to the results of the MINI questionnaire, one due to results of the AUDIT questionnaire, one due to concomitant medication use and one due to hypertension. Additionally, three participants withdrew informed consent after randomization: one due to an AE that occurred before drug intake and two due to logistical reasons.

**Figure 1:**
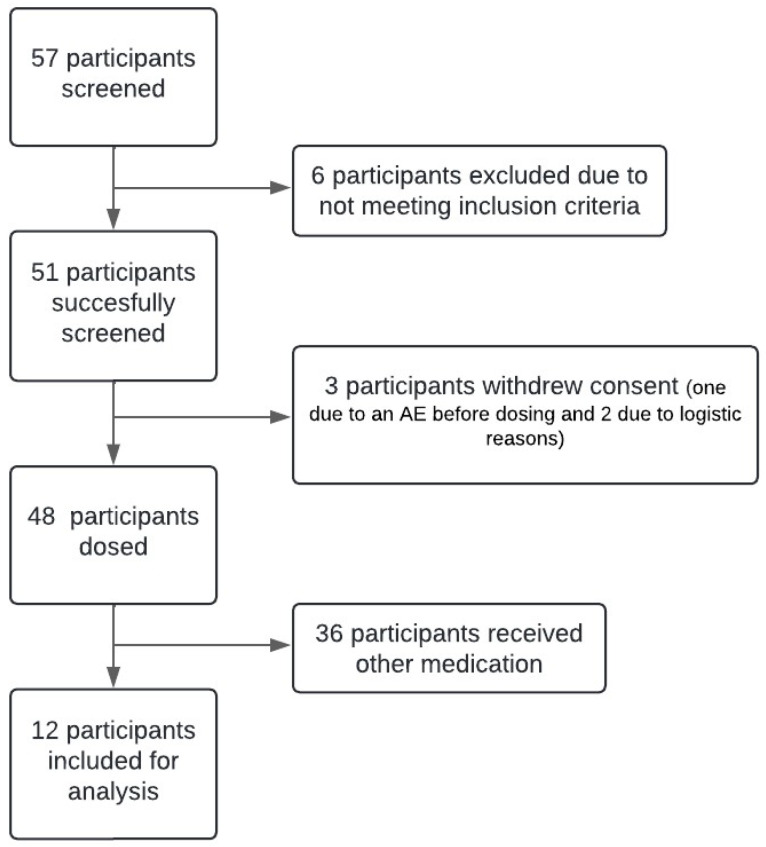
Flow chart of the study. A total of 57 participants were screened, of which 6 were excluded. Of these 51 participants, 3 participants withdrew consent. Of the remaining 48 participants, only the 12 participants that received 20 mg oxycodone were included for the analysis.

Of the 48 participants that completed the study, only the data of the 12 participants that received 20 mg of oxycodone on the lab visit was included in the current re-analysis; see Figure 1. Of these 12 participants, 4 were male and 8 were female with an age range of 19 to 69 years, with 2 participants being older than 50 years. The average age of the participants was 28 years, with a standard deviation of 16 years.

### 3.2. Oxycodone Pharmacokinetics

**Figure 2:**
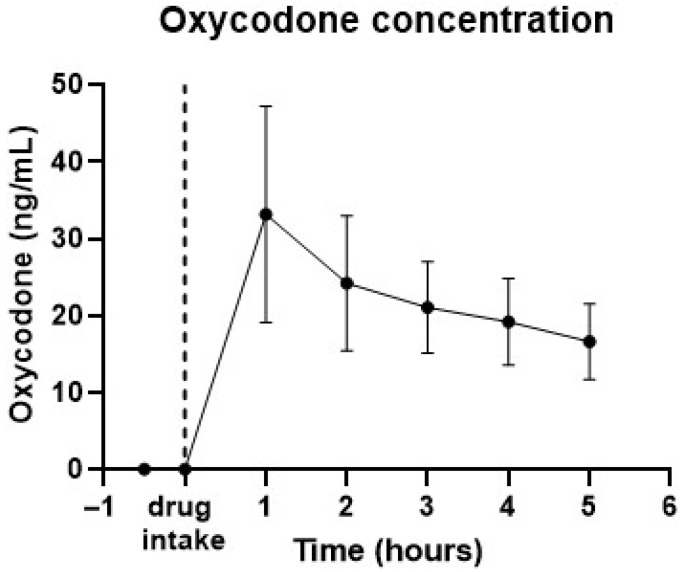
Pharmacokinetic data from 12 subjects taking 20 mg oral oxycodone. Graph shows average drug concentrations in time. Error bars represent standard deviations. Peak drug concentration was observed at 1 h after intake.

Venous blood samples were taken each hour to quantify drug levels. Figure 2 provides the pharmacokinetic data for 20 mg oral oxycodone, showing a peak concentration of approximately 33 ng/mL occurring 1 h post-dosing, with levels around 17 ng/mL 5 h after oxycodone intake.

### 3.3. Measuring Pupil Size and Light Quantity in the Room

Each participant performed 12 tests in the research unit when they were not under the influence of oxycodone and 16 tests after the intake of oxycodone. Of the tests in the research unit, half were made in dim-light conditions (approx. 50 lux) and half were made in bright-light conditions (approx. 500 lux). During the lab visits two participants performed notably more tests. This was due to initial rejection of their tests by the quality control analyses in the app, due to corneal arcus in these subjects. After updating the AI model [10], these tests were successfully included in the final analysis set. In the home period (7 days ± 2), participants performed three tests per day (approx. twenty-one tests). In the home environment, a total of 287 tests were performed. Of these tests, 184 were performed in dim-light conditions (below 140 lux, measured by the phone) and 103 in bright light (above 140 lux).

### 3.4. Pupil Size and Oxycodone Intake

**Figure 3:**
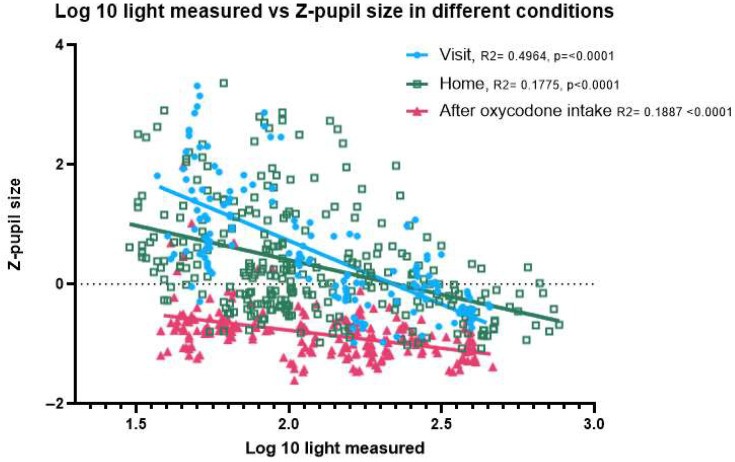
Correlation between light intensity and pupil size for different study conditions. Effect of oxycodone intake on pupil size in correlation to the amount of light in the room; home measurements separated from the measurements made during the study visits. This shows that after oxycodone intake, the pupil size is reduced (in both dark- and bright-light settings).

For measurements made in the same light condition, baseline pupil size is larger than that after oxycodone intake (Figure 3). In dark-light conditions (approx. 50 lux), the average z-pupil size before oxycodone intake is 0.799 and after oxycodone intake is −0.672. In bright-light conditions (approx. 500 lux), the average z-pupil size before oxycodone intake is −0.212 and after oxycodone intake is −0.985.

Figure 3 shows the pupil size before activating the torch of the mobile phone during the PLR test, expressed as z-scores. Without oxycodone (both home and visit before drug intake) the pupil size is highly variable and responsive to variation in ambient light conditions. After oxycodone intake the variability in pupil size is significantly reduced, and pupil size is on average smaller in all light conditions than without oxycodone. In dim-light conditions the pupil size without oxycodone (both at home and during the visit before drug intake) can be discriminated from the pupil size after intake of 20 mg oxycodone. In some participants, the net differences in pupil size under bright-light conditions, with and without oxycodone, were small. There was no significant difference in slope (*p* = 0.1083) or intercept (*p* = 0.6813), showing that the pupil size measurements taken at home closely matched those taken at the research unit prior to drug intake across all light conditions (Appendix B). We excluded two participants with corneal arcus, as the AI model occasionally misidentified the arcus ring as the pupil, leading to an increased variability in the data. Inclusion of the two participants rendered a small significant difference in slope, albeit small enough to have limited practical value for opioid classification (Appendix B).

### 3.5. Prediction of Oxycodone Use

**Figure 4:**
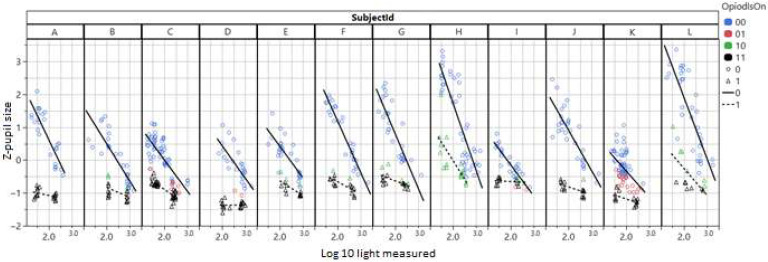
Dbase (z-pupil size) plotted vs. the log of ambient light measured with the mobile phone. Circles are before and triangles after administration of oxycodone. The classification results from a simple model with Dbase (z-pupil size) and Log light are shown in color. Black triangles are true positives and green triangles false negatives. Blue circles are true negatives and red circles false positives.

A model based solely on pupil size achieves an AUC of 0.92 and predicts oxycodone use with an accuracy of 80% for true positives and 89% for true negatives. When the model uses both pupil size and light intensity (measured by the phone), the model improves slightly to an AUC of 0.94 (95% CI 0.85–0.98). Oxycodone use can be identified in 82% of the cases, with a false positive rate of 9%. Of the measurements, 399 are true positive, 39 false positive, 41 false negative and 173 true negative. The false positives are mostly due to two participants with cornel arcus (Figure 4), because the AI model sometimes identifies the corneal arcus ring as the pupil which makes the measurement of the pupil size incorrectly large. For one participant (H), almost all tests after drug administration come back as ‘false negatives’, due to exceptionally large pupils, with this also being the case after drug administration (Figure 4).

For the remainder of participants, pupil size remained small during the 5 h following oxycodone intake and had not yet returned to normal after 5 h. The false negative tests were evenly distributed over the 5 h post-oxycodone intake, with an indication of a slight increase in negatives towards the end of the time period.

## 4. Discussion

In this randomized clinical study, we found that we can reliably detect oxycodone use by measuring pupil size with the use of the MPSES app installed on a consumer-grade smartphone. We show that even without individualization of the data, the AI model in the MPSES app accurately measures the pupil size, after which a logistic regression classifier is able to determine with an AUC of 0.94 if opioids have been used or not.

Drugs of abuse are known to affect pupil size and also ocular motility [14]. For opioids it is known that they cause constricted pupils, a reduced PLR, by binding to the µ-opioid receptor [15], while not causing horizontal or vertical nystagmus or lack of ocular convergence [14]. Incidences of opioids causing nystagmus are only found in the literature after epidural administration [16]. Therefore, nystagmus is not expected when examining the eyes of opioid users. However, patients with opioid use disorder have been shown to have a higher prevalence of convergence insufficiency [17]. Also, the pupil size of people using opioids for a longer time without increasing the dose have been reported to not significantly differ from people not using opioids [18]. This means that the dominant signature of opioid use is related to miosis; other effects on eyes may occur in special cases, and long-time use may affect the reaction pattern of the eye.

In 2021, it was estimated that there were 1 million high-risk opioid users in the EU [19]. Opioid use can be determined based on variable samples: urine, saliva, sweat and hair [20]. These all rely on bodily material, and the results are not always directly accessible. Due to the high amount of opioid use and abuse there is a need for a reliable easily accessible tool that gives a fast result, which would make it easier to identify opioid users. On top of that it could reduce the use of intrusive and costly urine tests [21]. A potential solution to this problem would be the use of non-invasive mobile phone-based eye scanning to quantify pupil size and thus opioid use [1]. The MPSES app looks for changes/abnormalities in non-convergence, nystagmus and pupillary light reflex. In this study we used the pupil size data from the pupillary light reflex, since that is found to be changed after opioid usage. Since light also influences the pupil size, the light conditions in which the pupil size is measured need to be taken into account. This can be seen in Figure 3, where the sober pupil size in bright-light conditions is on par with the oxycodone-constricted pupil in dim light, which is in line with observations made by others [11]. Omission of ambient light management will clearly increase the number of false results.

In our study it was found that measuring the correct pupil size in participants with corneal arcus presented a challenge for the AI model, resulting in a pupillograms with a lot of noise. This leads to difficulties in extracting key features, which potentially leads to false positives. Since the start of this study in 2023, the AI model has been retrained and updated (as mandated by medical device legislation) and now performs significantly better in handling eyes affected by corneal arcus, therefore reducing the chance of a false positive result.

We found a false negative rate of 18%, which may seem high. However, this is without individualization of the data, which potentially leads to false negatives among people with naturally large pupils. Individualization of the data will probably reduce the false negative rate. Currently in clinical practice, commonly used opioid urine immunoassays are generally not able to detect oxycodone or fentanyl usage [22] because of antibody specificity reasons. Hence, the MPSES method is capable of measuring opioid impairment irrespective of compound, although with an 18% false negative rate; this is in comparison to urine tests that detect a few selected opioids, disregarding other abundantly available opioids. Hence, what constitutes a high false negative rate must be seen in the perspective of the clinically used alternative.

Our PK data shows that the intake of 20 mg oral oxycodone caused a peak concentration at 1 h of around 33 ng/mL, at which time the peak effect of pupil constriction was also found. The oxycodone showed a half-life of around 4 h, which is in line with the known PK of oral immediate-release oxycodone [23,24]. The effect of this dosage on the pupil size is sufficient for detection of oxycodone use across various ambient light conditions up to 5 h after intake without the need for individualized data. Abuse-level dosages of opioid-type drugs have been reported to also be visible in eye characteristics the day after consumption [25]. The effects of misuse and abuse doses of opioids on eyes should therefore be visible for approximately 5–15 h.

This study has several limitations:

It is well known that the sober pupil size is dependent on, for example, age and cognitive load, such as attention and memory [26]. Other compounds, such as caffeine and nicotine, can influence pupil size [27,28]. These were not monitored during this study. This may have caused small variability in the data, especially during the home week since subjects were not able to smoke in the research unit. Taking these parameters into account in parallel with measurements could potentially improve the drug identification model in the future. In this study we used healthy participants that all received 20 mg oxycodone. This limited our ability to see potential effects of health issues in the measurements that we performed. Furthermore, our study provided limited insights into the effects of lower and higher dosages. After 5 h, at a concentration in blood of around 17 ng/mL (half of peak concentration), the MPSES app was still able to detect oxycodone usage, indicating that ingestion of a lower dose would probably also be detectable. Also we do not expect that a higher dosage would not cause the pupil size to reduce, but it would rather stay reduced for a longer time, as seen in [25]. Future research should consider looking into the effects of health problems, the effect of higher dosages and the combination of drugs. Finally, only one MPSES app was used in this study. This means that the sensor was developed based on the gold standard confirmed ingestion of oxycodone. The ability of the MPSES app to accurately depict the pupil or the eye movement has not been evaluated by comparison with other eye-scanning equipment. The reported findings are hence limited to the MPSES app used in this study. The sample size of 12 subjects is low for a clinical study, but since the effect of oxycodone on pupil size is known, the sample size is deemed sufficient to determine if the MPSES app can measure changes in pupils due to ingesting oxycodone.

## 5. Conclusions

This research shows that the Previct Drugs app can reliably detect opioid use for at least 5 h after intake, without requiring a sober reference when users perform the pupil measurement themselves.

## Figures and Tables

**Figure A3:**
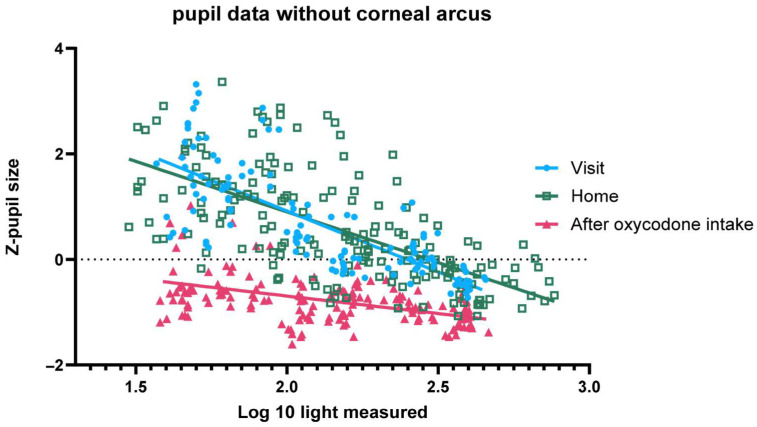
Z-pupil size (y-axis) versus log10 (ambient light) for ten individuals without corneal arcus, including data from home, sober at study site and under the influence of oxycodone at study site.

**Figure A4:**
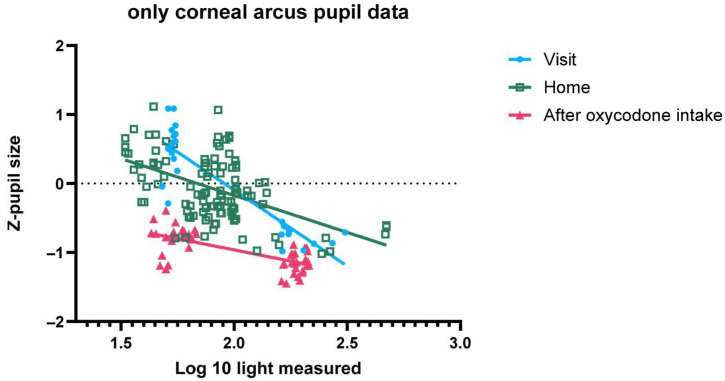
Z-pupil size (y-axis) versus log10 (ambient light) for two individuals with corneal arcus, including data from home, sober at study site and under the influence of oxycodone at study site.

## Data Availability

The original contributions presented in this study are included in the article; further inquiries can be directed to the corresponding author.

## Abbreviations

The following abbreviations are used in this manuscript:

AUC	Area under the curve
AI	Artificial Intelligence
MPSES	Mobile phone-based self-administered eye scanning
NC	Non-convergence
NY	Horizontal nystagmus
PK	Pharmacokinetic
PLR	Pupillary light reflex

## Appendix A

**Table A1:** Description of the eye-scanning procedures and the corresponding key features extracted from acquired data.

Procedure	Description	Key Features	Description
Pupillary light reflex (PLR)	Use voice guidance to instruct the person to (1) flip the phone to face the back side camera, and (2) position the camera adequately. Next, while looking straight into the smartphone camera with eyes wide open, illuminate the eyes with the flashlight for 4 s. Extract pupil sizes for both eyes over time from the collected video, estimate key features on each eye, and report average. The PLR procedure produced complete key feature data in 94% of the cases. Redness could be produced in 97% of the cases.	Dbase	Pupil size at start of illumination
		Dcon	Pupil size at maximum contraction
		Dend	Pupil size at end of illumination
		Latency	Time to first visible pupil size reaction
		Ctime	Time to Dcon
		MCV	Max contraction velocity, the largest negative slope during illumination
		PESC	Dend-Dcon
		MCA	Dbase-Dcon
		RMCA	MCA/Dbase
		Redness	Color of the sclera in CIELAB-A color coordinate
Non-convergence (NC)	Using voice guidance, ask the person to first look straight, and later to cross their eyes, all while facing the smartphone camera. Extract horizontal iris positions over time from collected video. The NC procedure produced key feature data in 94% of the cases.	NCdiff	[Distance between eyes when looking straight]–[Distance between eyes when crossing them]
Nystagmus (NY)	Using voice guidance, ask the person to first look straight, and later to look far to the side without turning the head, all while facing the smartphone camera. Extract horizontal iris position vs. time from collected video for the eye looking to the lateral side. If horizontal nystagmus occurs, it will be visible as a small peak in the graph of iris position vs. time. The NY procedure produced complete key feature data in 87% of the cases.	NYnumber	Number of peaks greater than a threshold per unit time in the trajectory of iris position over time while looking far to the side
		NYmass	Area of the peaks identified in Peak Counts

## Appendix B

Effect of oxycodone intake on pupil size in correlation to the amount of light in the room; home measurements are separated from the measurements made during the study visits and subjects with corneal arcus are visualized separately.

Figure A3 shows that the measurements made at home are not significantly different from the measurements made during the study visits before oxycodone intake in terms of slope (*p* = 0.1083) or intercept (*p* = 0.6813).

Figure A4 shows that there is more variation in the measurements made by the subjects with corneal arcus. This is due to the AI model sometimes identifying the corneal arcus ring as the pupil.
